# Immunochemotherapy achieved a complete response for metastatic adenocarcinoma of unknown primary based on gene expression profiling: a case report and review of the literature

**DOI:** 10.3389/fimmu.2023.1181444

**Published:** 2023-04-19

**Authors:** Jin Sheng, Hongming Pan, Weidong Han

**Affiliations:** ^1^ Department of Medical Oncology, Sir Run Run Shaw Hospital, School of Medicine, Zhejiang University, Hangzhou, China; ^2^ Key Laboratory of Biotherapy of Zhejiang Province, Sir Run Run Shaw Hospital, School of Medicine, Zhejiang University, Hangzhou, China

**Keywords:** cancer of unknown primary, immunochemotherapy, case report, gene expression profiling, metastatic adenocarcinoma

## Abstract

**Background:**

Cancer of unknown primary (CUP) is a malignant and aggressive tumor whose primary origin is still unknown despite thorough evaluation. CUP can be life-threatening with a median overall survival of less than 1 year based on empirical chemotherapy. Gene detection technology advances the driver gene detection of malignant tumors and the appropriate precise therapy. Immunotherapy has ushered in a new era in cancer therapy, changing the way advanced tumors, including CUP, are treated. Combined with comprehensive clinical and pathological investigations, molecular analysis of the original tissue and detection of potential driver mutations may provide therapeutic recommendations for CUP.

**Case presentation:**

A 52-year-old female was admitted to hospital for dull abdominal pain, with peripancreatic lesions below the caudate lobe of the liver and posterior peritoneal lymph nodes enlargement. Conventional biopsy under endoscopic ultrasonography and laparoscopic biopsy both revealed poorly differentiated adenocarcinoma based on immunohistochemical series. To help identify tumor origin and molecular characteristics, 90-gene expression assay, tumor gene expression profiling with Next-generation sequencing (NGS) method and Immunohistochemical expression of PD-L1 were employed. Although no gastroesophageal lesions discovered by gastroenteroscopy, the 90-gene expression assay yielded a similarity score and prompted the most likely primary site was gastric/esophagus cancer. NGS revealed high TMB (19.3mutations/Mb) but no druggable driver genes identified. The Dako PD-L1 22C3 assay IHC assay for PD-L1 expression revealed a tumor proportion score (TPS) of 35%. Given the presence of negative predictive biomarkers for immunotherapy, including adenomatous polyposis coli (APC) c.646C>T mutation at exon 7 and Janus kinase 1(JAK1), the patient received immunochemotherapy instead of immunotherapy alone. She was successfully treated with nivolumab plus carboplatin and albumin-bound nanoparticle paclitaxel for six cycles and nivolumab maintenance, which achieved a complete response (CR) maintained for 2 years without severe adverse events.

**Conclusions:**

This case highlights the value of multidisciplinary diagnosis and individual precision treatment in CUP. Further investigation is needed as an individualized treatment approach combining immunotherapy and chemotherapy based on tumor molecular characteristics and immunotherapy predictors is expected to improve the outcome of CUP therapy.

## Introduction

Cancer of unknown primary (CUP) is a malignant tumor whose primary lesion remains unknown despite extensive examination. It has been histologically confirmed as metastatic, accounts for 2-5% of all diagnosed cancers worldwide and is characterized by early and aggressive metastases (1).

CUP can be life-threatening and has an extremely poor prognosis, with a median overall survival of less than 1 year (2). CUP was roughly divided into a good prognosis group and a poor prognosis group, of which the poor prognosis group accounted for about 80% of the total and the poor prognosis group was dominated by empirical paclitaxel and platinum-based chemotherapy (3).

Due to the heterogeneous presentation of CUP, it is difficult to adequately answer important questions involving immunohistochemical testing, biological characteristics, tissue-of-origin molecular profiling, and novel therapies through traditional prospective randomized trials. However, in combination with comprehensive clinical and pathological investigations, molecular analysis of the original tissue and detection of potential driver mutations may provide therapeutic recommendations for CUP (4). It is important to formulate the best individualized treatment strategy combining clinical and molecular biological characteristics to improve the treatment outcome of CUP patients.

Here we describe a case of unfavorable CUP with multimodality diagnostic procedures and successfully treated with nivolumab plus carboplatin and albumin-bound nanoparticle paclitaxel for six cycles and nivolumab maintenance. The patient has achieved complete response (CR) which maintained for 2 years.

## Case presentation

**Figure 1:**
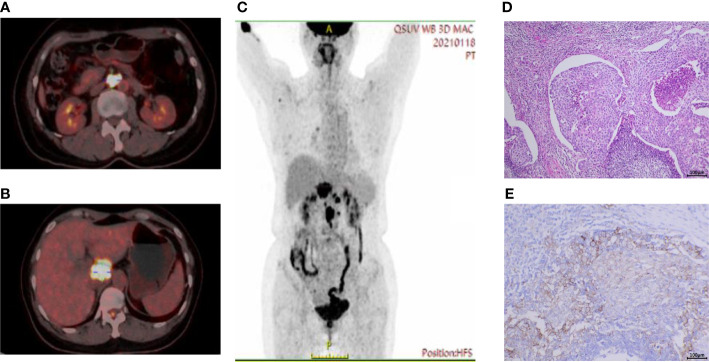
Baseline PET-CT imaging and pathologic results. **(A)** High uptake of fluorodeoxyglucose (FDG) in enlarged retroperitoneal lymph nodes and **(B)** peripancreatic lesions below the caudate lobe of the liver. **(C)** Coronal view showed multiple hypermetabolic lesions in the abdominal cavity. **(D)** Hematoxylin and eosin staining on hepatopancreatic lesions under endoscopic ultrasound showed poorly differentiated carcinoma. **(E)** The Dako PD-L1 22C3 assay immunohistochemistry assay for PD-L1 expression revealed that the tumor proportion score (TPS) was 35%. Scale bar = 100μm.

In January 2021, a 52-year-old female was admitted to the Medical Oncology Department at Sir Run Run Shaw Hospital for dull abdominal pain. The patient was previously healthy, denying hypertension, diabetes and other diseases. Physical examination found obvious pain under xiphoid process, no tenderness or rebound pain in other parts. CT enhancement of the abdomen showed peripancreatic lesions below the caudate lobe of the liver. The enlarged lymph nodes in the posterior peritoneum were suspected of malignancy. Peripheral blood tumor markers indicated normal AFP, CA199, CEA, and CA125 levels. There was only a slight increase in CA72-4 at initial diagnosis, and the baseline result was 13.96 U/ml (reference, <6.90 U/ml). Gastroenteroscopy did not reveal gastrointestinal space occupying lesions. Further endoscopic ultrasound was performed to puncture the hypoechoic mass in the hepatopancreatic space and the pathology indicated poorly differentiated carcinoma (**Figure 1D**). Immunohistochemical results on hepatopancreatic lesions by endoscopic ultrasound biopsy were CK-pan (+), CK7 (partial+), CK20 (-), P63 (-), P40 (-), CgA (-) and Syn (-).

To help identify the primary lesion, the patient underwent a positron emission tomography-CT (PET-CT) examination (**Figure 1**). The results showed that there were soft tissue mass shadows in the hepatic hilar region and pancreas head, with a maximum cross section of 35 mm x 34 mm, accompanied by increased FDG metabolism and SUVmax of 17.5. The local boundary with the pancreas neck is not clear. It also found multiple retroperitoneal lymph node enlargements with increased FDG metabolism, with SUVmax of 21.4, and the larger one with a short diameter of about 18 mm. These changes were considered to be associated with malignancy, pancreatic origin, or at least involvement.

**Figure 2:**
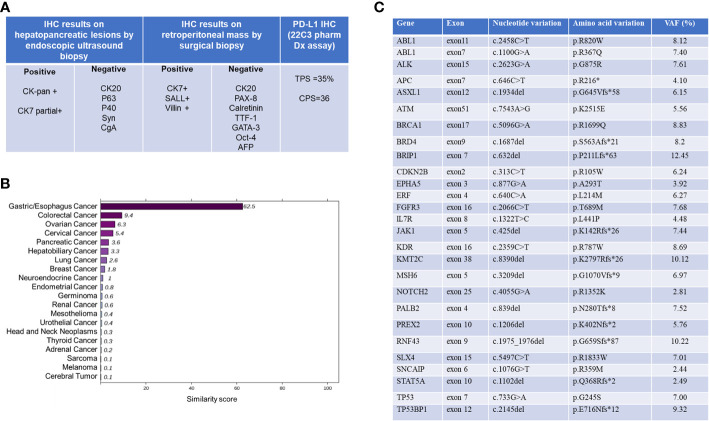
Summary of histopathological and molecular pathological information. **(A)** Pathological and immunohistochemical (IHC) results. **(B)** 90-gene tumor tissue traceability expression assay results. The maximum similarity score for tumor tissue traceability was 100. **(C)** Gene expression profile of tissue specimens analyzed by NGS. VAF, variant alle frequency. NGS, Next Generation Sequencing.

As the immunohistochemistry (IHC) results on hepatopancreatic lesions by endoscopic ultrasound biopsy were unable to determine the origin of this CUP case, this patient underwent MDT consultation and was recommended for minimally invasive laparoscopic surgical exploration and biopsy. General anesthesia with endotracheal intubation was performed in the supine position. After disinfection, an inverted L-shaped incision was made in the upper right abdomen, and the abdominal adhesion was separated layer by layer. There was slight adhesion in the abdominal cavity. The right paracolic sulci was opened, the right retroperitoneal space and hepatoduodenal ligament were separated, and masses in the portal vena cava space and beside the left renal vein, approximately 4cm x 4cm and 3cm x 2cm in size, were exposed. They were hard in texture and the lesion boundaries were unclear. No obvious abnormality was found in other organs of abdominal cavity. Pathological immunohistochemical series results based on surgical biopsies were positive for CK-pan, CK7, SALL4 and Villin. In addition, CK20, CK5/6, CgA, Syn, CD56, PAX-8, Calretinin, TTF-1, GATA-3, Oct-4 and AFP were negative. As summarized in **Figure 2A**, the final pathological diagnosis was a metastatic poorly differentiated carcinoma, combined with an immunohistochemical predisposition to digestive system origin.

Total RNA was isolated from FFPE tissue sections using an FFPE Total RNA Isolation Kit (Canhelp Genomics, Hangzhou, China). The 90-gene expression assay yielded a similarity score for each of the 20 tumor types based on formalin-fixed, paraffin-embedded cancer tissues. The top three predictions were gastric/esophagus cancer (62.5), colorectal cancer (9.4), and ovarian cancer (6.3), which indicated the most likely site was gastric/esophagus cancer (62.5, **Figure 2B**). The Dako PD-L1 22C3 assay immunohistochemistry assay for PD-L1 expression revealed a tumor proportion score of 35% (**Figure 1E**). NGS revealed MSS and high tumor mutation burden (TMB) as 19.3mutations/Mb. Besides, hot spot exon regions and some intron regions of more than 600 genes related to tumorigenesis and cancer development were sequenced by Illumina high-throughput sequencing platform. No druggable driver genes were found. The summary results are listed in **Figure 2C**.

To understand the pathways and biological functions involved, we further analyzed gene ontology (GO) and KEGG pathways. Finally, we identified the top 9 enriched GO terms as shown in **Supplementary Figure S1**. These findings indicated that gene expression profiling (GEP) results from this CUP may involve cell cycle regulation, protein tyrosine kinase activity, and homologous recombination.

**Figure 3:**
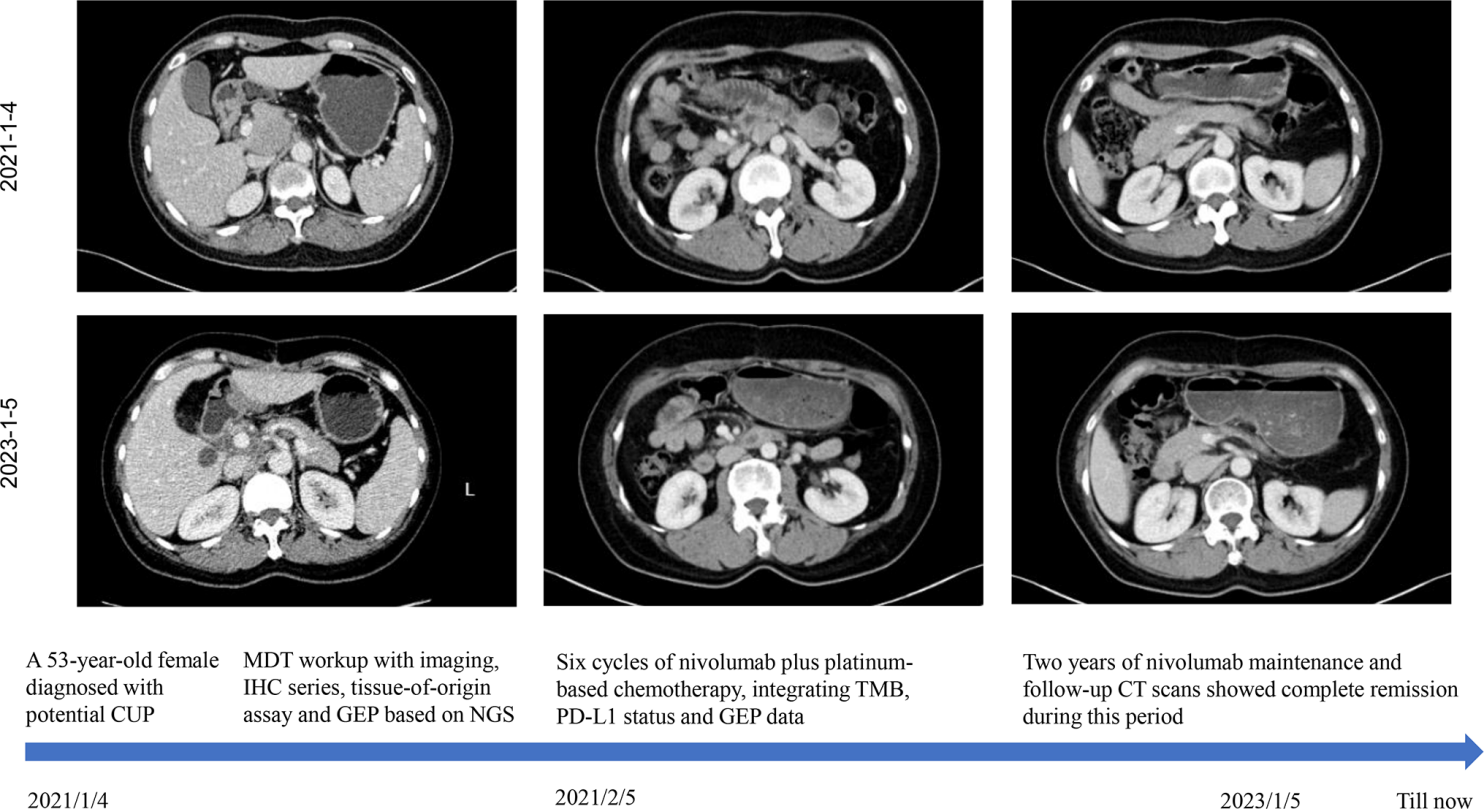
The patient’s clinical course schedule. Computed tomography scans of abdominal lesions at baseline (upper line) and after treatment (middle line) with nivolumab plus carboplatin and albumin-bound nanoparticle paclitaxel for six cycles and nivolumab maintenance. This case introduced an MDT workup with imaging, IHC series, tissue-of-origin assay, and NGS-based GEP. Immunochemotherapy incorporating TMB, PD-L1 status and GEP data benefited this patient with long-lasting response.

Given the presence of negative predictive biomarkers for immunotherapy, including adenomatous polyposis coli (APC) c.646C>T mutation at exon 7 and janus kinase 1 (JAK1) c.425del mutation at exon5, the patient is at risk of disease hyper-progression if given immunotherapy alone (**Figure 2C**). Therefore, she received nivolumab 200mg intravenously combined with albumin-bound nanoparticle paclitaxel 400mg intravenously and carboplatin 550mg intravenously, every 21 days, followed by regular nivolumab 200mg intravenously every 21 days for 2 years. Clinical and laboratory evaluations were required weekly during first treatment cycle then within 3days of each subsequent cycle. After six cycles of combination therapy, CT imaging indicated complete remission (**Figure 3**). Baseline elevated CA72-4 returned to normal after four cycles of treatment. With the addition of early supportive treatment, the patient’s chemotherapy-related nausea and vomiting and myelosuppression were satisfactorily controlled. Only mild nausea, fatigue, and grade 1 anemia occurred during combination therapy. The patients were satisfied with the therapeutic effect and showed good compliance. There were no significant adverse events during nivolumab maintenance, and regular follow-up CT scans showed that baseline lesions maintained complete remission within 2 years, without any previously unappreciated gastroesophageal lesions found.

## Discussion

Here we describe a case of unfavorable CUP with multimodality diagnostic procedures including tumor imaging and extensive review of pathology. With the help of tissue-origin-sequencing and gene expression profiling, the patient received the tailored combination strategy. After six cycles of nivolumab plus carboplatin and albumin-bound nanoparticle paclitaxel, she achieved complete response (CR) which maintained for 2 years during nivolumab maintenance.

One widely accepted definition of CUP is defined by the National Comprehensive Cancer Network (NCCN) as histologically proven metastatic malignant tumors whose primary site cannot be identified during pretreatment evaluation. Median survival was less than 1 year when empirical chemotherapy was used as the primary treatment for CUP. For CUP with poor prognosis, both the National Comprehensive Cancer Network (NCCN) and ESMO guidelines recommend platinum, taxanes, or gemcitabine-based empiric chemotherapy and participation in clinical trials. Objective response rate (ORR) of different chemotherapy regimens ranged from 10% to 50%, and median overall survival (OS) ranged from 4 to 13 months (5–7).

The development of molecular tumor profiling in CUP patients over the past decade has helped predict the location of tumor origin. Researchers have discovered genes and specific expression patterns related to the origin of tumor tissue (8, 9). Several novel assays of gene expression have been shown to be effective in predicting the primary source of a wide range of tumor types, supporting its diagnostic utility for molecular classification in difficult-to-diagnose metastatic cancer (10, 11). Using gene expression profiling, the “90-gene expression assay,” a real-time PCR assay, was recently developed to identify 21 common cancer types. It calculates the test sample similarity score for each cancer type, reflecting how closely the test sample’s gene expression pattern is similar to the global gene expression pattern of a known cancer. The tumor type with the highest similarity score was considered the predicted tumor type. In a retrospective cohort of 609 clinical samples, the gene expression assay was 90.4% accurate for primary tumors and 89.2% accurate for metastatic tumors (10). In addition, the gene expression assay was able to provide insightful predictions of primary tumors in 82.3% of patients in a real-world cohort of 141 CUP patients (116/141) (10). These data support the diagnostic utility of the 90-gene expression assay for molecular classification in difficult-to-diagnose metastatic cancer.

Compared with immunohistochemistry, the detection method based on gene expression profile improves the sensitivity and specificity of tumor origin tissue, but its guiding value for treatment is still controversial (12, 13). A meta-analysis included 5 original studies with a total of 1114 patients, of whom 454 received site-specific therapy and 660 received empiric therapy. Site-specific therapy was not significantly associated with improvements in PFS or OS compared to empirical therapy. Subgroup analyses, however, found that site-specific therapy significantly increased OS performance in the high-precision predictive analysis subgroup (HR 0.46, 95% CI 0.26-0.81, P = 0.008). Site-specific therapy also improved survival for patients with more responsive tumor types compared to patients with less responsive tumor types (14).

The development of gene detection technology promotes the discovery of the driving genes of malignant tumors and the corresponding precise therapy. Patients with CUP may potentially benefit from comprehensive genomic profiling-informed treatment. A multi-omics study of 97 CUP patients revealed frequent actionable genomic alterations with high levels of evidence among CUP patients, and better clinical outcomes in patients with high versus low degrees of precision matching. However, the molecular landscape of each patient was unique (15). Although it has been reported that 30% of CUPs have clearly targetable therapeutic genetic alterations (16), no pharmacologically tractable molecular alteration was found by NGS on tissue samples in our case.

Immunotherapy, represented by immune checkpoint inhibitors, has ushered in a new era of cancer treatment, changing the paradigm of treatment for many patients with advanced tumors. The approval of immunocheckpoint inhibitors has improved the prognosis of patients with CUP. Programmed cell death-1 (PD-1) inhibitor pembrolizumab is the world’s first approved anti-tumor drug for use regardless of tissue origin. In 2020, the FDA accelerated the approval of pembrolizumab for the treatment of advanced solid tumor patients who have progressed after previous treatment and no alternative treatment plan available, and whose tumor mutation load is more than 10 mutations/million bases. It is worth mentioning that the efficacy of single immunotherapy agents for CUP is limited. A phase II clinical study of pembrolizumab in advanced uncommon tumors demonstrated preliminary antitumor activity, in which 22 CUP patients who received at least one course of pembrolizumab had a progression-free survival rate of 33% and an ORR of 23% at 27 weeks (17). In another Phase II study in CUP, the effect of pembrolizumab was further investigated (18). Poorly differentiated carcinoma was present in 14 (56%) of the 25 eligible and evaluable patients (29 enrolled). Prior to enrolment, patients had already received a median of two lines of treatment. With acceptable safety profiles, the ORR of pembrolizumab was 20.0% (95% CI: 6.8–40.7), median PFS and OS were 4.1 (95% CI: 3.1–5.1) and 11.3 (95% CI: 5.5–17.1), respectively (18). In addition, Nivolumab’s efficacy in treating subtypes of CUP with poor prognosis was evaluated in the NivoCUP study, a multi-center Phase II study, which found that the ORR of CUP patients who had previously received chemotherapy was 22.2%, and the median OS was 15.9 months (19). In light of these results, nivolumab was also approved as the first immunological agent for the treatment of CUP in Japan. Moreover, nivolumab was more effective in the subgroup with high expression of programmed cell death ligand 1 (PD-L1), high tumor mutation load, and MSI-H (19). This suggests that it may be possible to further improve the efficacy of immunotherapy in CUP patients by identifying biomarkers that benefit from immunotherapy. Using a multiplex testing approach, 28% of CUP carried one or more predictive biomarkers (MSI-H, PD-L1 and/or TML-H) to the immune checkpoint blockade (20). The CUP patient we report is an microsatellite (MS) stable type, but the TMB result was 19.3 mutations/Mb with moderately positive for PD-L1 (22C3, TPS=35% and CPS=36), indicating a possible benefit from immunotherapy. When practitioners considered systemic treatment options, the presence of negative predictive biomarkers (JAK1 loss of function mutations for our patient) to immune checkpoint inhibitors also needs to be take into account to avoid tumor hyper-progression.

The strategy of nivolumab combined with chemotherapy in the neoadjuvant phase of NSCLC, advanced NSCLC or advanced gastroesophageal malignancy has been fully demonstrated to be effective and well-tolerated (21–24). Moreover, pembrolizumab combined with platinum-based chemotherapy has also achieved good efficacy in a previous reported CUP case (25). Since PD-L1 TPS was less than 1%, that patient received six cycles of pembrolizumab plus platinum and pemetrexed. However, tumor cells in that case were positive for thyroid transcription factor-1 (TTF-1) and cytokeratin 7, indicating the possibility of NSCLC origin.

Targeted therapy and immunotherapy guided by molecular biological detection are the main directions of future research (26). It is hoped that treatment based on molecular biological characteristics can surpass platinum-based empirical chemotherapy and improve the prognosis for CUP (16). To date, this case is the known one with the longest follow-up time and the most comprehensive diagnosis and treatment of CUP. Our case highlights the importance of the multidisciplinary diagnosis and individual precision treatment based on integrated tissue-origin-sequencing with molecular profiling information. It should be noted that this is a case study and further research and clinical trials are needed to confirm these findings.

Last but not least, the experience in this case may have implications for reassessing the diagnosis and treatment of CUP. Current guidelines for systematic treatment of CUP include conventional chemotherapy regimen, directed molecular targeted therapy based on driver genes, and immunotherapy recommendations for patients with MSI-H or dMMR, high expression of TMB or PD-L1 (27). However, existing treatment guidelines for systematic treatment of CUP may overlook the strategic value of immunotherapy-based combination therapy. Although molecular profiling of tumor tissue and NGS based on solid tumor biopsy or liquid biopsy as an alternative have been recommended for therapeutic decision-making, predictors of immunotherapy efficacy found by tests, especially negative predictors, should also be fully considered in CUP treatment decisions. Immunochemotherapy, antiangiogenic therapy, and dual immunotherapy are worthy of future exploration in CUP. In the future, the optimal individualized treatment strategies and the design of clinical trials combined with molecular biological characteristics are expected to prolong the survival of CUP patients.

## Conclusion

Our case suggests that the individualized treatment plan combined with immunotherapy and chemotherapy based on tumor molecular characteristics and immunotherapy predictors is expected to improve the outcome of CUP treatment and deserves further study.

## Data availability statement

The original contributions presented in the study are included in the article/**Supplementary Materials**. Further inquiries can be directed to the corresponding author.

## Ethics statement

The studies involving human participants were reviewed and approved by The Institutional Review Board (IRB) of Sir Run Run Shaw Hospital, School of Medicine, Zhejiang University. The patients/participants provided their written informed consent to participate in this study.

## Author contributions

JS: Conceptualization, Methodology and Writing- Original draft preparation. WH: Supervision, Writing- Reviewing and Editing. HP: Writing- Reviewing and Editing. All authors contributed to the article and approved the submitted version.

## Supplementary material

Supplementary Figure 1 GO enrichment plot and KEGG pathway analysis based on gene expression data. The count indicates the number of genes related to the enriched GO pathway. The color of the bar denotes the -log10 (p.adjust). The top 3 terms of enriched biological process (BP) were DNA recombination, negative regulation of cell cycle phase transition, and negative regulation of cell cycle. The following cellular composition (CC) terms significantly correlated with this GEP were DNA repair complex, condensed chromosome, chromosomal region. Furthermore, molecular function (MF) enrichment analysis showed that gene expression profiling (GEP) was significantly correlated with protein tyrosine kinase activity, protein serine/threonine/tyrosine kinase activity, transmembrane receptor protein tyrosine kinase activity. Interestingly, KEGG pathway analysis demonstrated pathways related to homologous recombination, Fanconi anemia pathway, and microRNAs in cancer.
