# Supplementary figures and images for: Immunochemotherapy achieved a complete response for metastatic adenocarcinoma of unknown primary based on gene expression profiling: a case report and review of the literature
Source: Front Immunol. 2023 Apr 19;14:1181444. doi: 10.3389/fimmu.2023.1181444 (PMC10154565; doi:10.3389/fimmu.2023.1181444)

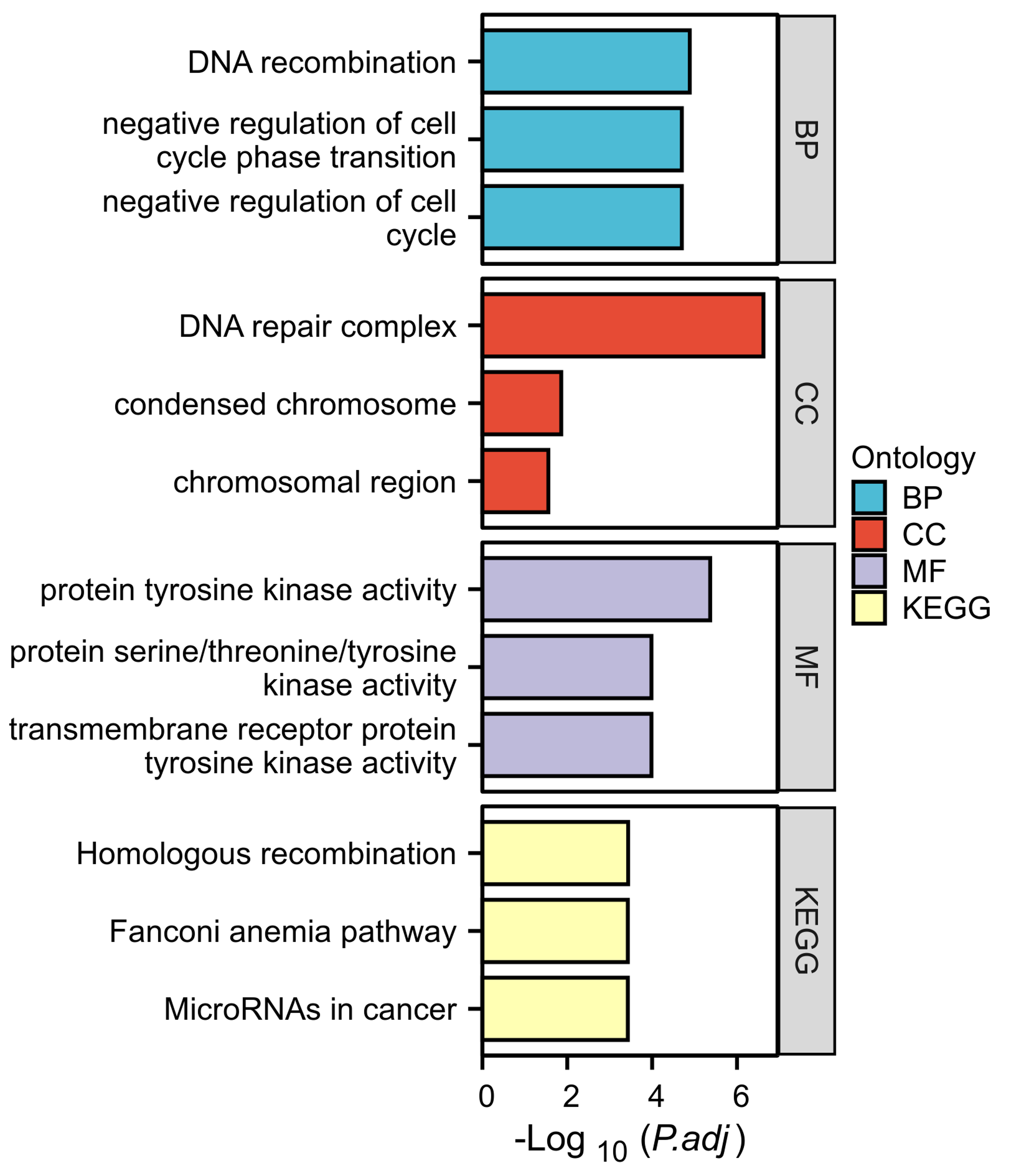

Supplement: Supplementary Figure 1 — GO enrichment plot and KEGG pathway analysis based on gene expression data. The count indicates the number of genes related to the enriched GO pathway. The color of the bar denotes the -log10 (p.adjust). The top 3 terms of enriched biological process (BP) were DNA recombination, negative regulation of cell cycle phase transition, and negative regulation of cell cycle. The following cellular composition (CC) terms significantly correlated with this GEP were DNA repair complex, condensed chromosome, chromosomal region. Furthermore, molecular function (MF) enrichment analysis showed that gene expression profiling (GEP) was significantly correlated with protein tyrosine kinase activity, protein serine/threonine/tyrosine kinase activity, transmembrane receptor protein tyrosine kinase activity. Interestingly, KEGG pathway analysis demonstrated pathways related to homologous recombination, Fanconi anemia pathway, and microRNAs in cancer. [file Image_1.tif]
